# A Brief Memoir of Sir Wm. Blizard, Knt., F.R.S. L. and E., Surgeon and Vice-President of the London Hospital

**Published:** 1836-07

**Authors:** 


Art. X.—*A brief Memoir of* Sir William Blizard, Knt. F.R.S. L. and E. *Surgeon and Vice President of the London Hospital. Read before the Hunterian Society, 7th October,* 1835; *with additional particulars of his Life and Writings.* By William Cooke, Member of the Royal College of Surgeons, &c. &c. London. Pp. 67.

Sir William Blizard had outlived his contemporaries. A life prolonged to more than ninety years had introduced him to a new generation, and he retained the desire to act with those whose attainments and feelings were not of his time. To most of the living practitioners his name is chiefly known as that of a tenacious elderly gentleman, celebrated for alarming the candidates for college honours in Lincoln's Inn Fields. He was the son of an auctioneer at Barnes Elms, and was born in 1743. His appointment to the London Hospital, long the scene of his active labours, took place in 1780; and in conjunction with Dr. Maclaurin, a Scotch physician, he established the first regular medical school in connexion with such an institution, in 1785. His biographer records that he suggested, and first practised, the operation of tying the superior thyroideal artery in bronchocele; that he was one of the first surgeons who secured the subclavian; and introduced the practice of large and repeated bleedings in fractures of the ribs. The last operation which he performed in public was in 1827, when he was eighty-four years of age. His clinical merits are stated to have been considerable, but his professional reading appears to have been very limited. Perhaps the circumstance most honorable to his professional memory is, that he was thought worthy of the high praise of John Abernethy, who had been his pupil, and who spoke of him, in his Inquiry into Hunter's Theory of Life, in terms of grateful recollection. His services in the London Hospital called forth the strongest expressions of acknowledgment from the governors on several occasions. In the prosperity of the College of Surgeons he seems ever to have felt a strong interest. He was twice president; he thrice delivered the Hunterian oration; and, on retiring from his lectures at the hospital, he presented his anatomical and pathological collection to the college, amounting to nearly nine hundred specimens. We gather also from Mr. Cooke's account of his connexion with this chartered body, that a part of his services consisted of a resolute resistance to certain reforms. His benevolence, however, was warm and active, and manifested by continual exertions of public and of private charity. Like many men of advanced age, he gradually abandoned the political sentiments which in early life he had warmly advocated. The energy of his temperament, which had sometimes endangered his life in prolonged anatomical labours, led him to become a member of the London Military Foot Association in 1780; and at a later period he was a zealous lieutenant-colonel of the London Volunteers. He was much interested, also, in police and prison reforms; and one of his most valuable publications related to the improvement of hospitals and other charitable institutions, which was translated into German. It should be added, also, that throughout a long life of usefulness, his efforts were always directed by a humble belief in the superintendence of a power which beheld all good efforts with approbation. With characteristic professional confidence, he submitted to the operation for cataract when ninety-one years old: it was performed by Mr. Lawrence with the happiest results. With an equally characteristic attachment to the Muse, Sir William commemorated his recovery of sight by an ode.

We cannot say that Mr. Cooke's Memoir is very well compiled, or pay any compliment to it as a composition; but there are many particulars in it not undeserving of the student's perusal.

